# RPS3a Over-Expressed in HBV-Associated Hepatocellular Carcinoma Enhances the HBx-Induced NF-κB Signaling via Its Novel Chaperoning Function

**DOI:** 10.1371/journal.pone.0022258

**Published:** 2011-08-16

**Authors:** Keo-Heun Lim, Kyun-Hwan Kim, Seong Il Choi, Eun-Sook Park, Seung Hwa Park, Kisun Ryu, Yong Kwang Park, So Young Kwon, Sung-Il Yang, Han Chu Lee, In-Kyung Sung, Baik L. Seong

**Affiliations:** 1 Department of Biotechnology, College of Life science and Biotechnology, Yonsei University, Seoul, Korea; 2 Department of Pharmacology, IBST, Konkuk University School of Medicine, Seoul, Korea; 3 Department of Internal Medicine, IBST, Konkuk University School of Medicine, Seoul, Korea; 4 Department of Anatomy and Center for Cancer Research and Diagnostic Medicine, IBST, Konkuk University School of Medicine, Seoul, Korea; 5 Research Institute of Medical Sciences, Konkuk University, Seoul, Korea; 6 Department of Internal Medicine, University of Ulsan College of Medicine, Asan Medical Center, Seoul, Korea; 7 Translational Research Center for Protein Function Control, Yonsei University, Seoul, Korea; University of Hong Kong, Hong Kong

## Abstract

Hepatitis B virus (HBV) infection is one of the major causes of hepatocellular carcinoma (HCC) development. Hepatitis B virus X protein (HBx) is known to play a key role in the development of hepatocellular carcinoma (HCC). Several cellular proteins have been reported to be over-expressed in HBV-associated HCC tissues, but their role in the HBV-mediated oncogenesis remains largely unknown. Here, we explored the effect of the over-expressed cellular protein, a ribosomal protein S3a (RPS3a), on the HBx-induced NF-κB signaling as a critical step for HCC development. The enhancement of HBx-induced NF-κB signaling by RPS3a was investigated by its ability to translocate NF-κB (p65) into the nucleus and the knock-down analysis of RPS3a. Notably, further study revealed that the enhancement of NF-κB by RPS3a is mediated by its novel chaperoning activity toward physiological HBx. The over-expression of RPS3a significantly increased the solubility of highly aggregation-prone HBx. This chaperoning function of RPS3a for HBx is closely correlated with the enhanced NF-κB activity by RPS3a. In addition, the mutational study of RPS3a showed that its N-terminal domain (1–50 amino acids) is important for the chaperoning function and interaction with HBx. The results suggest that RPS3a, via extra-ribosomal chaperoning function for HBx, contributes to virally induced oncogenesis by enhancing HBx-induced NF-κB signaling pathway.

## Introduction

Chronic infection of hepatitis B virus (HBV) is one of the major causes of liver cirrhosis and hepatocellular carcinoma (HCC) development [1], [2]. The hepatitis B virus X protein (HBx) is a key factor in HCC development during HBV infection [3], [4] and is detected in the majority of HBV-related HCC patients' tissues [5]. A number of studies have shown that HBx promotes cell survival and stimulates signaling pathways including Ras/MAPK cascades, Wnt/β-catenin signaling and PI-3 kinase/Akt pathways, which are closely related with tumorigenesis [3], [4]. These upstream signaling changes by HBx ultimately lead to the transactivation of transcription factors such as NF-κB, which influence the cell proliferation and eventually cancer development [3], [6]–[11].

Recently, extensive studies on differential over-expression gene profiles in HBV-induced HCC tissues were performed [12], [13]. Interestingly, among those over-expressed genes, a number of ribosomal subunit components (RPS20, RPL27a, RPLP0, RPL8, RPL31, RPL37a, RPS24, RPS27a, RPL21, RPL35a, RPS8, RPS37a, RPS3a, RPL36a) were identified [13]. The mRNA levels of ribosomal subunits in HBV-induced HCC tissues were 5.7∼20.5-fold higher as compared with the non-tumor normal tissues. Among them, RPL36a was reported to be associated with cellular proliferation in HCC [14]. RPL26 regulates the translation and induction of p53 after DNA damage [15]. These studies suggest that such ribosomal proteins are involved in cell transformation rather than their classical role in protein synthesis. Still, the role of the over-expressed ribosomal proteins in the HBV-associated HCC and their relationship with viral proteins remains largely unknown.

Among the over-expressed genes, ribosomal protein S3a (RPS3a) exhibited 5.7 fold higher expression in HBV-induced HCC patient tissues [13]. RPS3a, a component of the ribosomal small subunit (40S) [16], shows high sequence homology in mammalian cells and is localized in both the nucleus and cytoplasm [17]. RPS3a also has multiple biological functions unrelated with the ribosome. Previously known as Fte-1 (v-*fos* transformation effector gene), RPS3a is an inducing factor for oncogenic cellular transformation of Rat-1 cells by *v-fos* [18], [19]. In addition, RPS3a is highly expressed in most tumors including hepatocellular carcinoma [13], [20] and other cancers [21]–[25]. Even though the over-expression of RPS3a alone does not directly induce cell transformation, RPS3a is closely related with abnormal cell proliferation and transformation via extra-ribosomal activity [18], [26], [27]. Importantly, it is reported that Epstein-Barr virus (EBV)-induced B cell transformation leads to the up-regulation of RPS3a. Furthermore, EBV-encoded nuclear antigen, EBNA-5 is reported to bind RPS3a, suggesting a role for EBV-induced B cell transformation [17]. On the other hand, RPS3a has also been described as a regulator of apoptosis and transcription factors [27]–[29]. RPS3a interacts with a transcription factor, GADD153, and modulates erythropoiesis [30]. To date, a number of studies have shown that RPS3a is associated with cell proliferation, differentiation and tumor formation. However, there is no report on the underlying mechanism for the multi-functional roles of RPS3a.

Here, we explored the possibility that RPS3a can enhance the HBV-induced tumorigenesis by interacting with the viral oncogenic HBx protein. Of note, HBx is highly aggregation-prone and is expressed as punctate form in eukaryotic cells [31]–[34] and as insoluble inclusion bodies in *E. coli* [35]–[37]. The highly aggregation-prone tendency of HBx suggests that there might be some cellular factors that assist the maintenance of HBx solubility for its function. Consistent with this idea, molecular chaperones have known to be involved in cancer development by stabilizing oncogenic proteins [38]–[40]. In addition, it has recently been reported that soluble macromolecules such as ribosomal proteins and RNA can function as chaperones [41]–[45]. Thus, we, for the first time, investigated whether RPS3a has a novel chaperoning activity for viral oncoprotein HBx. The present study will give new insights into the role of cellular proteins over-expressed in HBV-associated HCC.

## Results

### RPS3a enhances the HBx-induced NF-κB signaling

**Figure 1:**
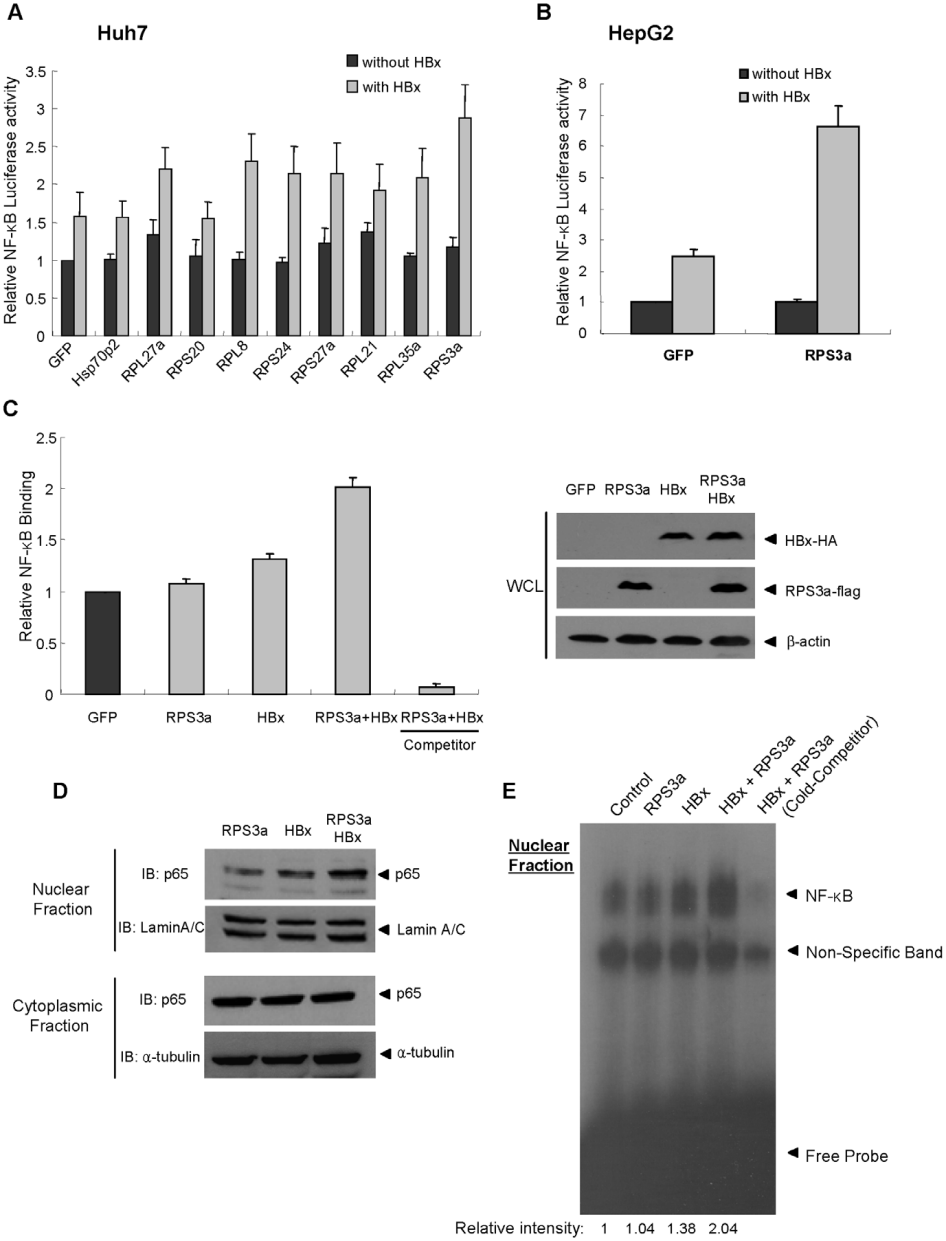
RPS3a enhances the HBx-induced NF-κB Signaling. (**A–B**) Relative NF-κB activity in Huh7 and HepG2 liver cells after co-transfection of pNF-κB-Luc(0.25 µg), pEGFP(0.4 µg) (control plasmid) and the indicated plasmid(0.4 µg) with/without pEG-HBx(0.4 µg). (**C**) ELISA of NF-κB activation in nucleus. Using nuclear extracts (4 µg), the binding between activated NF-κB subunit in nucleus and plate-bound NF-κB consensus DNA oligomer was measured by chemiluminescence (left). Free unbound NF-κB consensus DNA oligomer was used as a competitor. Total expression levels of HBx and RPS3a in whole cell lysate (WCL) were determined by indicated antibodies (right). (**D**) Western blot analysis of p65 translocation in Huh7 cells. Lamin A/C and α-tubulin were used for loading controls of nuclear and cytoplasmic fractions, respectively. The loading amount in cytoplasmic fractions is 5-fold dilution than that of the nuclear fractions. (**E**) Electrophoretic Mobility Shift Assay (EMSA). Separated nuclear extracts (3 µg) were subjected to *in vitro* binding with [P^32^]-labeled NF-κB consensus DNA oligomer probe. pEGFP vector was used as control vector. As a cold competitor, excess (30-fold) non-labeled NF-κB consensus oligomer was used. Relative binding intensity was calculated by using densitometry.

The profiles of the cellular proteins that are over-expressed in the HBV-associated HCC tissues have been reported [13]. Interestingly, many of these were ribosomal proteins. HBx has been well established to activate the transcription factor NF-κB (1.5∼2-fold) through regulation of cancer-related signaling pathways [3], [8]–[11]. Therefore, we investigated whether the over-expressed cellular proteins in the HBV-related HCC tissues can influence the oncogenic activity of HBx. For this purpose, we initially selected and cloned 18 over-expressed cellular genes in the HBV-associated HCC tissues, most of which are ribosomal proteins. After the co-tranfections of the cloned genes with HBx, their effects on the HBx-mediated NF-κB activation were tested using the luciferase reporter system. Through the first screening (data not shown), 9 genes were selected for further study. Among them, as shown in Figure 1A, seven genes (RPL27a, RPL8, RPS24, RPS27a, RPL21, RPL35a and RPS3a) out of 9 significantly upregulated the HBx-induced NF-κB activity in Huh7 cells. In particular, RPS3a was observed to enhance the HBx-induced NF-κB activity most efficiently. This observation was more prominent in HepG2 cells (Figure 1B).

To verify the enhancing ability of RPS3a on the HBx-induced NF-κB signaling directly, we analyzed the nuclear translocation of NF-κB subunits using NF-κB ELISA method and the electrophoretic mobility shift assay (EMSA) as described in Materials and methods. The nuclear NF-κB ELISA showed that the expression of RPS3a itself alone did not affect the NF-κB activity, whereas in the presence of HBx, RPS3a synergistically enhanced the NF-κB activity. However, the over-expression of RPS3a did not affect the total level of HBx (Figure 1C). The nuclear translocation of p65 was further confirmed by western blot analysis after co-transfection of HBx and RPS3a. The coexpression of HBx and RPS3a significantly promoted the nuclear localization of p65, whereas the total amount of p65 in cytoplasm was slightly decreased based on the ratio of p65/tubulin (Figure 1D). Finally, we performed the EMSA to verify the enhancing ability of RPS3a. The data clearly show that RPS3a hyperactivates the HBx-induced NF-κB signaling (Figure 1E). The overall results of the luciferase reporter assay, nuclear translocation of p65 and EMSA demonstrate that RPS3a over-expressed in HBV-associated HCC tissues enhances the HBx-induced NF-κB signaling in liver cells.

### RPS3a is over-expressed in HBV-associated HCC tissues

**Figure 2:**
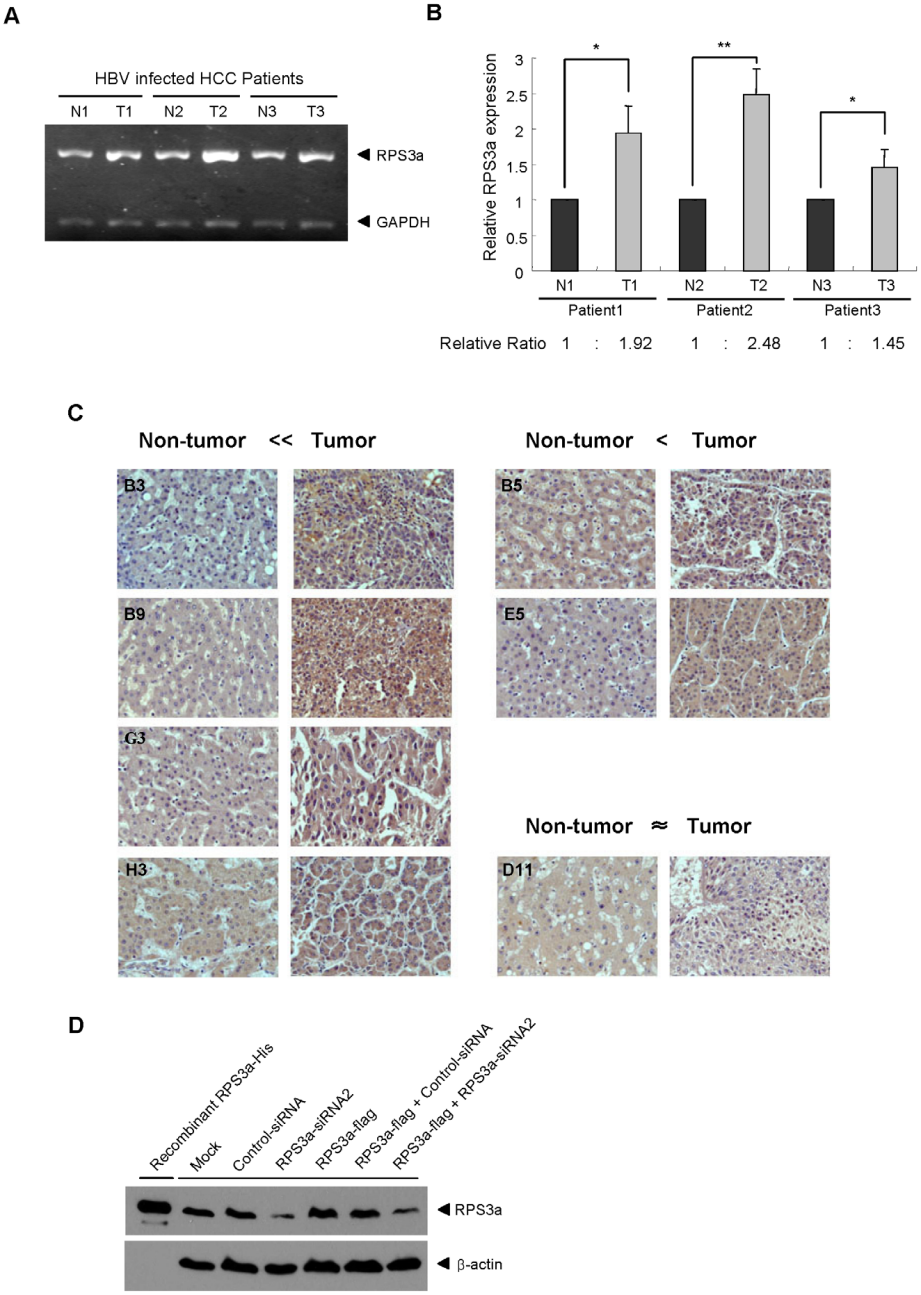
Expression of RPS3a in HBV-associated hepatocellular carcinoma tissues. (**A**) Detection of endogenous RPS3a mRNA levels in the tumor and non-tumor regions of HBV-associated HCC tissues. Total RNA was extracted from tumor or non-tumor tissues of three HBV-caused HCC patients. Using total RNA extracts (2 µg) and oligo dT primer, cDNA was synthesized. N and T represent the non-tumor tissues and tumor tissues, respectively. (**B**) Relative expression ratio of RPS3a mRNA between the tumor and non-tumor tissues of HBV-associated HCC. Data were calculated by three independent experiments (* P<0.05 and ** P<0.001). Data represent the intensity ratio of RPS3a band after normalization to GAPDH in the Bio-1D image analysis software. (**C**) Representative immunohistochemistry data of endogenous RPS3a protein expression in the HBV-associated HCC tissues. The difference of RPS3a expression between non-tumor and tumor tissues was determined by visual inspection under microscope (magnification ×400) and assigned on the top of the panels. See Table 1 for full information. (**D**) Generation and validation of human RPS3a antibody. Human RPS3a protein was produced in *E. coli* and the rabbit antibody was generated. The specificity of the generated antibody was validated by knock-down and over-expression of RPS3a in Huh7 cells. Huh7 cells were harvested at 72 hr after transfection.

Because the previous profile report of over-expressed proteins in the HBV-associated HCC was monitored mainly using Northern blot analysis [13], it is further necessary to investigate the over-expressed protein levels in the HBV-associated HCC tissues. First, we compared the relative mRNA levels of RPS3a between tumor and non-tumor regions in the tissues from three HBV-associated HCC patients (IBR approved), using semi-quantitative reverse transcription PCR. Consistent with the previous results, the RPS3a expression is significantly higher in tumor regions of HBV-associated HCC tissues than in peripheral non-tumor regions (Figure 2A and 2B). To further confirm the levels of RPS3a protein expression in tissues, we produced recombinant RPS3a in *E. coli*, and generated the anti-RPS3a antibody in rabbits. The specificity of anti-RPS3a was confirmed using knock-down and over-expression systems (Figure 2D). Immunohistochemical analysis of 20 cases of HBV-positive HCC tissues, paring non-tumor and tumor tissues was carried out using anti-RPS3a antibody. The 70% (14 cases) of HBV-positive HCC tissues showed considerably over-expression of RPS3a in tumor regions compared with the corresponding peripheral non-tumor regions (Table 1). The representative immunohistochemistry data show that the endogenous level of RPS3a protein is significantly higher in the tumor regions of HBV-associated HCC than in non-tumor regions (Figure 2C).

**Table 1:** Expression level of RPS3a and information of tissue samples.

Tissue No.	Sex	Age	HBV	HCV	RPS3a expression
A7	F	51	+	−	NT≪T
B3	M	43	+	N.D	
B9	M	42	+	−	
D1	M	60	+	−	
E3	M	49	+	−	
F9	M	53	+	−	
G3	M	61	+	−	
H3	M	50	+	−	
B5	M	51	+	+	NT<T
B7	M	59	+	−	
E1	F	40	+	−	
E5	F	63	+	−	
E11	M	37	+	−	
H11	M	51	+	N.D	
C1	F	65	+	−	NT≈T
D11	M	59	+	−	
F5	M	43	+	−	
G5	M	56	+	−	
G11	M	54	+	−	
H9	M	69	+	N.D	

N.D: Not Deterimed; NT: Non-Tumor; T: Tumor.

### Over-expression of ribosomal proteins influence the expression patterns of HBx

**Figure 3:**
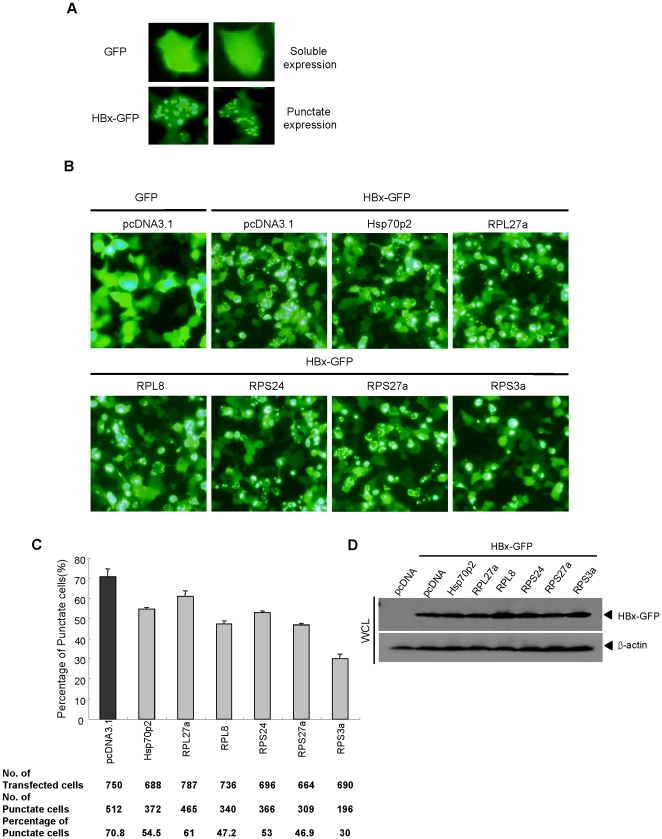
The expression of ribosomal proteins reduces the punctate expression of HBx in liver cells. (**A**) The representative patterns of GFP and HBx-GFP expression in liver cells. Huh7 cells were transfected with 2 µg of pEGFP or pEG-HBx-GFP plasmid in 6-well plates. After 36 hr, the expression pattern of each protein was examined by fluorescence microscopy (magnification ×400). (**B**) Reduction of punctate expression of HBx-GFP by ribosomal proteins. pEG-HBx-GFP (2 µg) and indicated plasmids(2 µg) were coexpressed in Huh7 cells. pEGFP or pcDNA3.1 plasmids were used as control (magnification ×200). (**C**) Number of cells showing punctate expression was counted under fluorescence microscopy. Ratios of punctate cells were calculated and presented by bar graph. (**D**) Total expression levels of HBx-GFP in whole cell lysate (WCL) were determined by anti-GFP antibody after co-transfection of indicated ribosomal subunits and pEG-HBx-GFP.

To understand how the over-expressed ribosomal proteins in HCC tissues can influence the HBx-induced NF-κB activity, we first examined the expression pattern of HBx using HBx-GFP fusion protein in Huh7 cells after co-transfection with ribosomal proteins. Previous studies have demonstrated that the expression pattern of HBx-GFP fusion protein is largely punctate with granule-like insoluble aggregates in the cytoplasm [32]. The typical pattern of HBx-GFP expression is shown in Figure 3A. Interestingly, the over-expression of ribosomal proteins did not alter the total expression level of HBx-GFP (Figure 3D), but influenced the expression pattern of HBx-GFP protein (Figure 3B). Among the ribosomal proteins, RPS3a resulted in a dramatic decrease in its punctate expression when co-transfected with HBx-GFP plasmid. The percentage of cells showing punctate expression was about 30% when RPS3a was co-transfected, whereas that in control pcDNA3.1 transfection was about 70% (Figure 3C). We found that the reduction of punctate expression was correlated with the enhanced activity of HBx-induced NF-κB signaling (Figure 1A). These findings imply that RPS3a is associated with the soluble expression of HBx in a functional form, which is also relevant to HBx activity such as NF-κB signaling.

### RPS3a enhances the HBx-induced NF-κB signaling via its chaperoning activity

**Figure 4:**
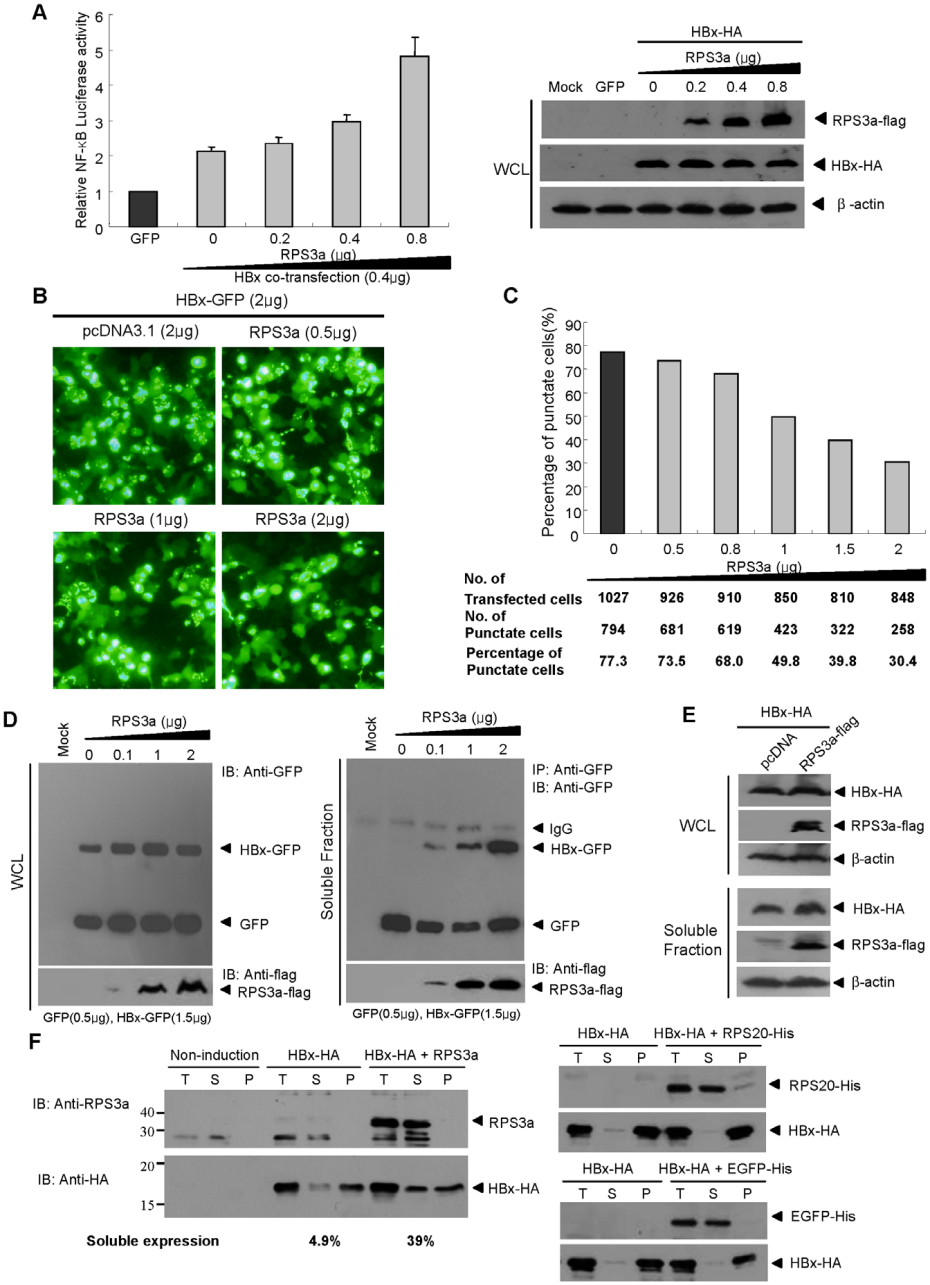
RPS3a enhances HBx-induced NF-κB signaling via its chaperoning activity. (**A**) Dose-dependent activation of NF-κB by RPS3a. The pEG-HBx (0.4 µg) was co-transfected with increasing amounts of RPS3a and pNF-κB-Luc (0.25 µg) in Huh7 cells. Total DNA amounts (1.5 µg) were adjusted using pEGFP in 12-well plates. Total expression levels of RPS3a and HBx in whole cell lysate (WCL) were determined by anti-flag and anti-HA antibodies, respectively (right). (**B–C**) Decrease of punctate expression of HBx-GFP by RPS3 in a dose-dependent manner. Huh7 cells were co-transfected with pEG-HBx-GFP (2 ug) and increasing amounts of RPS3a. The amount of total transfected DNA was normalized using pcDNA3.1. Representative patterns of HBx-GFP expression at 36 hr post-transfection are presented in panel B (magnification ×200). The number of cells showing punctate expression of HBx-GFP was counted under fluorescence microscopy and the ratio of punctate cells was calculated in panel C. (**D**) Soluble expression of HBx-GFP in cytoplasm by RPS3a. The pEG-HBx-GFP (1.5 µg) was co-transfected with increasing amounts of pcD-RPS3a-flag. The pEGFP(0.5 µg) was transfected as a control for transfection and detection by GFP antibody. At 96 hr post-transfection, cells were lysed and cytoplasmic soluble fraction was immunoprecipitated with anti-GFP antibody (right). As a control, the whole cell lysate (WCL) before centrifugation was analyzed for the total expression of HBx-GFP (left). Note the expression ratio of HBx-GFP/GFP. (**E**) Soluble expression of HBx in cytoplasm by RPS3a. After co-transfection of pcD-HBx-HA and pcD-RPS3a-flag, the whole cell lysate (WSL) and the soluble fraction (supernatant of WCL after centrifugation) were analyzed by western blot analysis. (**F**) RPS3a enhances the soluble expression of HBx in *E. coli* expression system. *E. coli* cells were co-transformed with pT7-HBx-HA and pBad-RPS3a-His plasmids. After culture and co-induction by IPTG and L-arabinose, total cell extracts were obtained by sonication and divided into fractions using centrifugation at 12000 rpm (T, S and P represent total cell extracts, supernatant, and pellet fraction, respectively). The expression pattern of HBx was analyzed by western blot. Soluble expression was calculated by densitometry. As random protein controls, pBad-RPS20-His, another ribosomal subunit and pBad-EGFP-His plasmids were used for co-transformation with pT7-HBx-HA in *E. coli* cells. Both RPS20 and EGFP were detected by anti-penta His antibody (Qiagen).

To elucidate the role of RPS3a in more detail, we assessed the effects of RPS3a on both HBx-induced signaling and the soluble expression of HBx. As shown in Figure 4A and 4B, the NF-κB activity and the soluble expression of HBx were significantly enhanced in proportion to RPS3a, whereas the total expression levels of HBx were constant (Figure 4A). Consistent with NF-κB activity (Figure 4A), the punctate expression of HBx was also greatly decreased in a RPS3a dose-dependent manner (Figure 4B and 4C). To show that RPS3a enhances the soluble expression of HBx in cytoplasm, we measured the amount of soluble HBx in the cytoplasmic fraction by both immunoprecipitation and western blot analysis after co-transfection with RPS3a. The immunoprecipotation result clearly showed that the soluble expression of HBx was increased by RPS3a in a dose-dependent manner (Figure 4D, right), whereas the total expression level of HBx-GFP was not changed (Figure 4D, left). Furthermore, we observed a similar result by direct western blot analysis of soluble fraction and whole cell lysate obtained after co-transfection of RPS3a and HBx-HA (Figure 4E). However, the over-expression of another ribosomal subunit that did not affect the HBx-induced NF-κB activation (Figure 1A), RPS20, did not increase the soluble expression of HBx (data not shown). The results suggest that RPS3a affects the solubility of HBx rather than its stability.

To find more direct evidence, we investigated a solubility-enhancing ability of RPS3a for HBx using the *E. coli* expression system [45], [46]. The expression of RPS3a-His and HBx-HA were controlled by L-arabinose and IPTG, respectively. Expression of HBx alone resulted in almost insoluble product (Soluble fraction: 4.9%); however, upon coexpression with RPS3a, the soluble fraction of HBx protein greatly increased to 39% (Figure 4F, left). As the control experiments, we coexpressed the RPS20 or EGFP with HBx and observed no increase of soluble expression of HBx (Figure 4F, right). These data demonstrate that this phenomenon is not restricted to mammalian cells, suggesting that the chaperone function of RPS3a on HBx is provided irrespective of the cellular environment. Taken together, these results suggest that the chaperoning effect of RPS3a on HBx is likely responsible for the increased functional activity of HBx.

### Knock-down of RPS3a abolishes the RPS3a-mediated NF-κB enhancement by HBx

**Figure 5:**
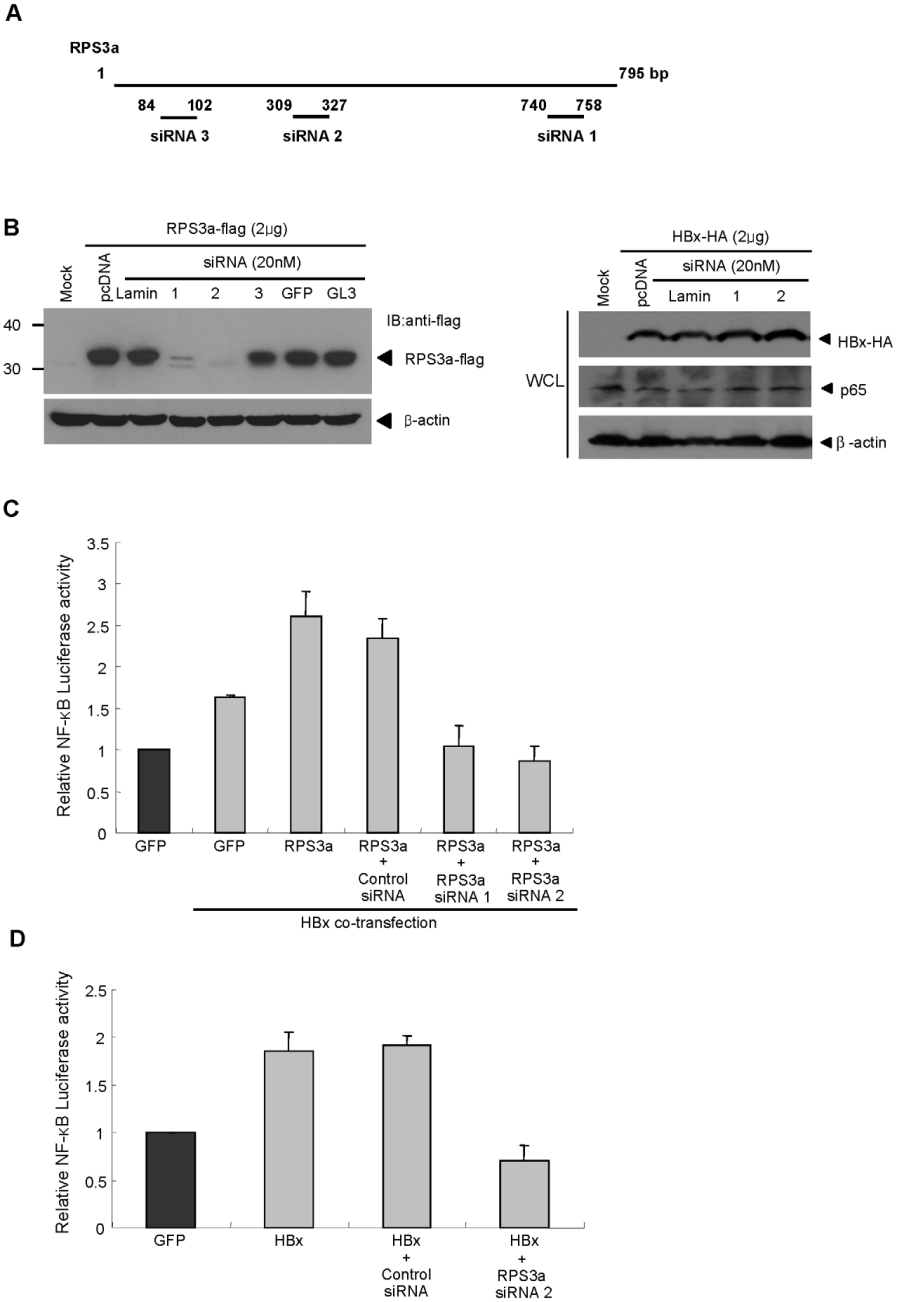
Knock-down of RPS3a abolishes RPS3a-mediated NF-κB enhancement by HBx. (**A**) A schematic representation of siRNA design on RPS3a gene. (**B**) The effect of three siRNA candidates on RPS3a, HBx and endogenous p65 expression. Cells were co-transfected with pcD-RPS3a-flag (or pcD-HBx-HA) and each RPS3a siRNA candidate (siRNA1, 2, 3), and harvested after 72 hr post-transfection. siRNAs against lamin, GFP, luciferase (GL3) were used as control siRNAs. Expression levels of proteins were detected by indicated antibodies. (**C**) Effect of RPS3a knock-down on NF-κB activity. The plasmids of pcD-RPS3a (0.4 µg), pEG-HBx (0.4 µg), and pNF-κB-Luc (0.25 µg) were co-transfected with the RPS3a siRNA1 or siRNA2. Lamin siRNA was used as a control siRNA. (D) Effect of endogenous RPS3a on HBx-mediated NF-κB activation. NF-κB activity was determined at 30 hr post co-transfection of pNF-κB-Luc and indicated plasmids with/without siRNAs in Huh7 cells. Note that knock-down of the endogenous RPS3a lowered the HBx-induced NF-κB activity to the background level.

To further investigate the effect of RPS3a on HBx-mediated NF-κB signaling, we performed the knock-down experiment using RNA interference. We designed the siRNAs against RPS3a and found that the RPS3a expression was greatly decreased by siRNA1 and 2 (Figure 5A and 5B, left). The treatment of RPS3a siRNAs did not affect the total expression level of HBx and p65 (Figure 5B, right). The treatment with siRNA1 or 2 under RPS3a and HBx coexpression completely abolished the RPS3a-mediated NF-κB enhancement, whereas the control siRNA had no effect (Figure 5C). Of particular interest is the level of decrease in the NF-κB activity by siRNAs. The treatment of siRNA decreased the RPS3a-mediated NF-κB enhancement by HBx commensurate to the basal level (GFP alone), which was much less than that of HBx alone. These data imply that the endogenous RPS3a is associated with the HBx-induced NF-κB activation. To confirm the involvement of endogenous RPS3a in HBx-mediated NF-κB activation, we determined the HBx-induced NF-κB activity under the knock-down of endogenous RPS3a by siRNA. The knock-down of endogenous RPS3a was confirmed as described in Figure 2D. Under this condition, the NF-κB activity was decreased blow the basal level (Figure 5D), confirming that the endogenous RPS3a has an important role for the HBx-induced NF-κB activation.

In particular, because the expression of RPS3a is abundant in HCC tissues (Table 1) [13], the effect of over-expression of RPS3a in HCC cell lines is expected to be marginal due to saturation in the cytoplasm. Therefore, the results from knock-down of RPS3a by siRNA would provide more compelling evidence than the over-expression.

### Identification of the domain of RPS3a necessary for chaperoning function on HBx

**Figure 6:**
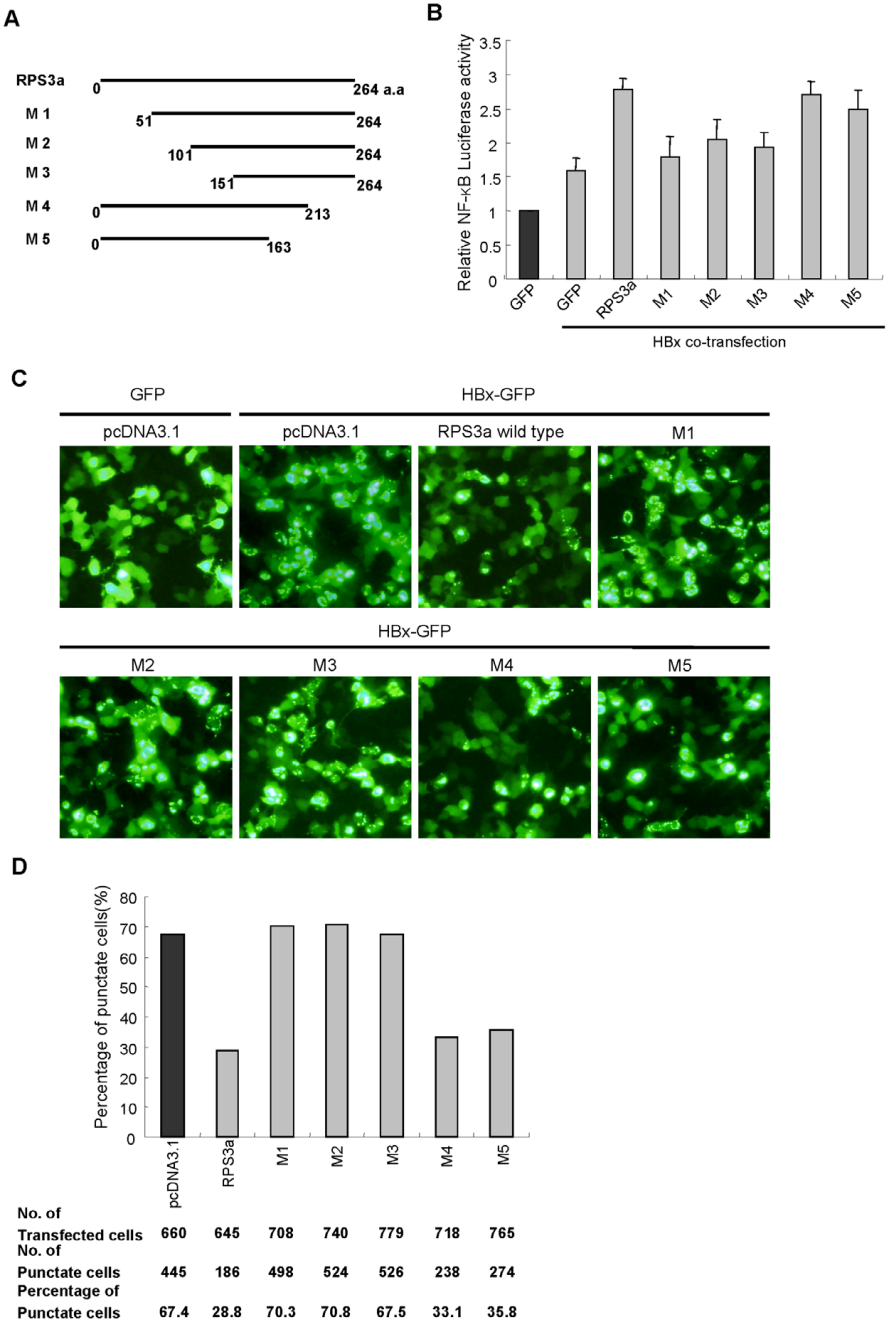
The N-terminal domain of RPS3a is critical for NF-κB hyperactivation and the soluble expression of HBx. (**A**) Construction of RPS3a deletion mutants. (**B**) The N-terminal domain of RPS3a is responsible for HBx-induced NF-κB activity. After co-transfection with wild-type or deletion mutants of RPS3a (0.4 µg) and pEG-HBx (0.4 µg) in Huh7 cells, the relative NF-κB activity was measured. (**C**) The N-terminal region of RPS3a is responsible for the punctate expression of HBx. After 36 hr co-transfection with pEG-HBx-GFP (2 µg) and wild-type or mutant RPS3a (2 µg), the expression pattern of HBx-GFP protein was examined by fluorescence microscopy (magnification ×200). (**D**) Number of cells showing punctate expression was counted under fluorescence microscopy. Ratio of punctate cells was calculated and presented by bar graph.

**Figure 7:**
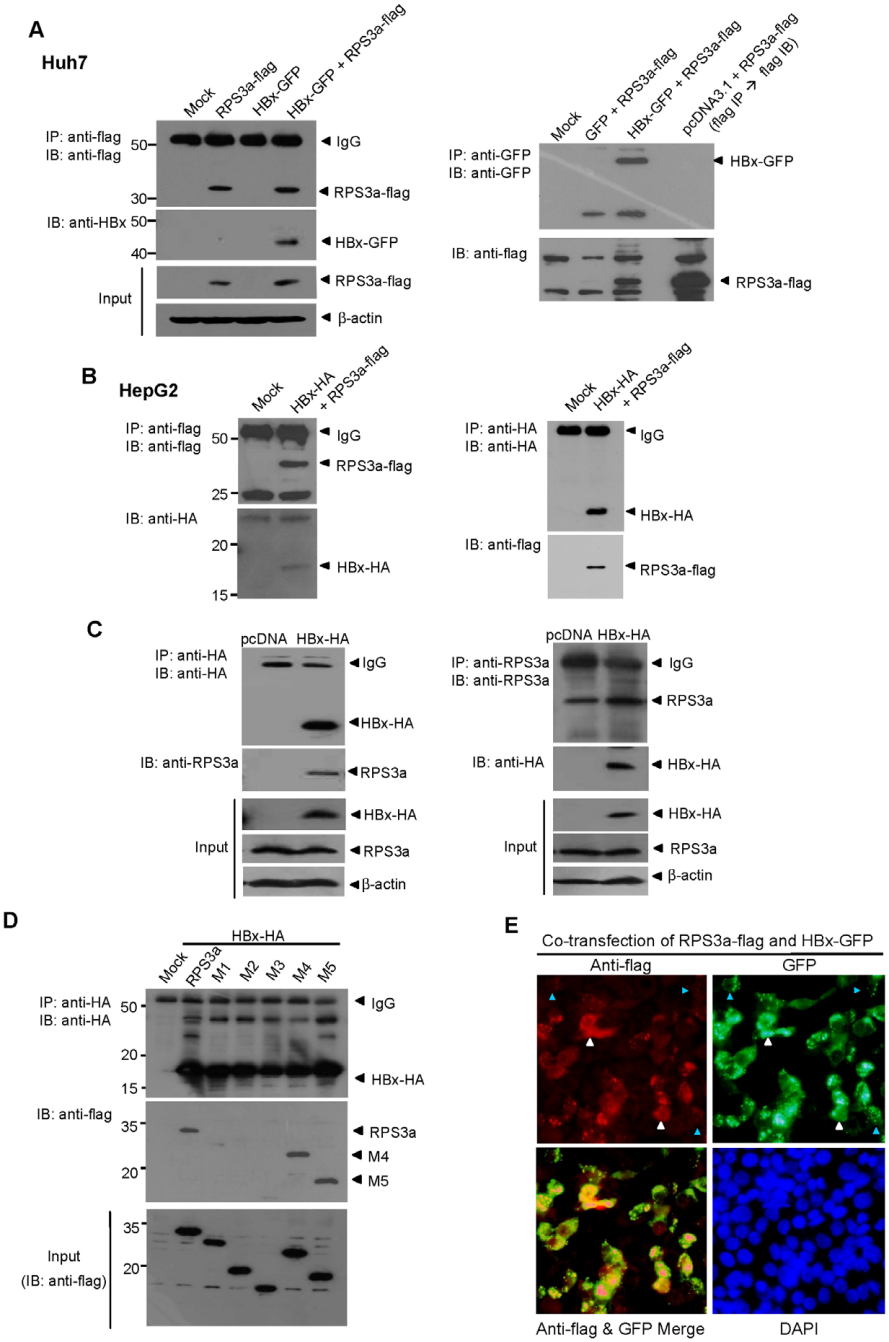
RPS3a interacts with HBx via its N-terminal domain and co-localizes in liver cells. (**A**) RPS3a physically interacts with HBx in liver cells. pEG-HBx-GFP and pcD-RPS3a-flag were transfected in Huh7 cells. At 96 hr post-transfection, cell lysates were immunoprecipitated with anti-flag antibody (left) and anti-GFP antibody (right). Western blot analysis was performed using indicated antibodies. After stripping, each membrane was treated with anti-HBx antibody (left) and anti-flag antibody (right). As a positive control for identifying the RPS3a-flag band, immunoprecipitation was performed with anti-flag followed by western blot using the same antibody. (**B**) Interaction of HBx with RPS3a in HepG2 cells without GFP-fusion. The pcD-HBx-HA and pcD-RPS3a-flag were co-transfected in HepG2 cells and the immunoprecipitation and western blot were performed as indicated. (**C**) Interaction of HBx with endogenous RPS3a in Huh7 cells. The pcD-HBx-HA was transfected in Huh7 cells and immunoprecipitation and western blot were performed as indicated. (**D**) Identification of RPS3a binding domain critical for HBx interaction. Wild-type pcD-RPS3a-flag (2 µg) or pcD-mutants RPS3a-flag (M1∼M5) (2 µg) were co-transfected with pcD-HBx-HA (2 µg) in Huh7 cells. Aliquots of total lysates were used for western blot analysis (lower panel). The remaining lysates were immunoprecipitated with anti-HA antibody and detected by anti-flag antibody. After stripping, the membrane was reblotted by anti-HA antibody. (**E**) HBx and RPS3a co-localize in transfected liver cells. Huh7 cells were co-transfected with pEG-HBx-GFP (2 µg) and pcD-RPS3a-flag (2 µg). After 24 hr, immunocytochemistry analysis was performed using anti-flag antibody. White and blue triangles indicate the cells with high and low expression of RPS3a, respectively. Note that low expression of RPS3a is linked with punctate expression of HBx.

To identify the domain of RPS3a that is responsible for the chaperoning activity on HBx, we generated several deletion mutants of RPS3a as described in Figure 6A, and the expression of these mutants was confirmed by western blot analysis (Figure 7D). Using these deletion mutants, we performed the HBx-induced NF-κB activation assay. All the N-terminal deletion mutants (M1 to M3) significantly decreased the HBx-induced NF-κB hyperactivation, while the C-terminal deletion mutants (M4, M5) showed no significant change in the NF-κB hyperactivation compared to the wt RPS3a (Figure 6B).

In addition, the effect of RPS3a mutants on the soluble expression of HBx was analyzed using fluorescence microscopy. The N-terminal deletion mutants lost the ability to solubilize the puntate expression of HBx-GFP, in contrast with the C-terminal mutants (M4, M5) (Figure 6C and 6D). These solubility-enhancing effects of RPS3a mutants are well correlated with their HBx-induced NF-κB hyperactivation (Figure 6B). These findings demonstrate that the N-terminal domain of RPS3a (amino acids 1–50) is responsible for the RPS3a-mediated chaperoning function on HBx.

### RPS3a interacts with HBx via its N-terminal domain

Generally, the chaperoning function of a protein is mediated by the protein-protein interaction between the molecular chaperone and target protein. Therefore, we investigated whether the chaperoning activity of RPS3a is mediated by the physical interaction between RPS3a and HBx. We performed the co-immunoprecipitation assay after co-transfection of RPS3a-flag and HBx-GFP in Huh7 cells. The data clearly show that RPS3a interacts with HBx in the cytoplasm (Figure 7A). To further verify the interaction between RPS3a and HBx, we conducted the co-immunoprecipitation assay using RPS3a-flag and HBx-HA constructs in HepG2 cells; these assays also confirmed that RPS3a interacts with HBx in cytoplasm (Figure 7B). In addition, to verify the association between RPS3a and HBx in physiologically relevant environment, we examined whether the endogenous RPS3a can interact with HBx. We confirmed that the endogenous RPS3a interacts with HBx (Figure 7C), supporting the result of Figure 5D.

The above results in Figure 6 demonstrate that the N-terminal domain of RPS3a is responsible for the chaperoning function on HBx, allowing us to predict that this domain is necessary for RPS3a binding with HBx. The RPS3a deletion mutants (Figure 6A) were subjected to co-immunoprecipitation assay after co-transfection with HBx. The expression levels of the deletion mutants in Huh7 cells were similar; however, only mutants M4 and M5 were bound with HBx (Figure 7D). These data demonstrate that the N-terminal 50 amino acids are responsible for RPS3a binding with HBx. The results are well correlated with previous data of the chaperone activity of RPS3a on HBx (Figure 6). Taken together, the N-terminal 50 amino acids of RPS3a are responsible for the chaperoning activity via binding with HBx.

Finally, we confirmed the interaction between RPS3a and HBx using co-localization assays (Figure 7E). As predicted, RPS3a was co-localized with HBx. Notably, the weak expression of RPS3a (blue triangles) is correlated with punctate expression of HBx, whereas the cells over-expressing RPS3a show soluble expression of HBx (white triangles).

### RPS3a enhances HBx-induced NF-κB signaling in the context of a replication-competent full HBV genome via its chaperoning function for HBx

**Figure 8:**
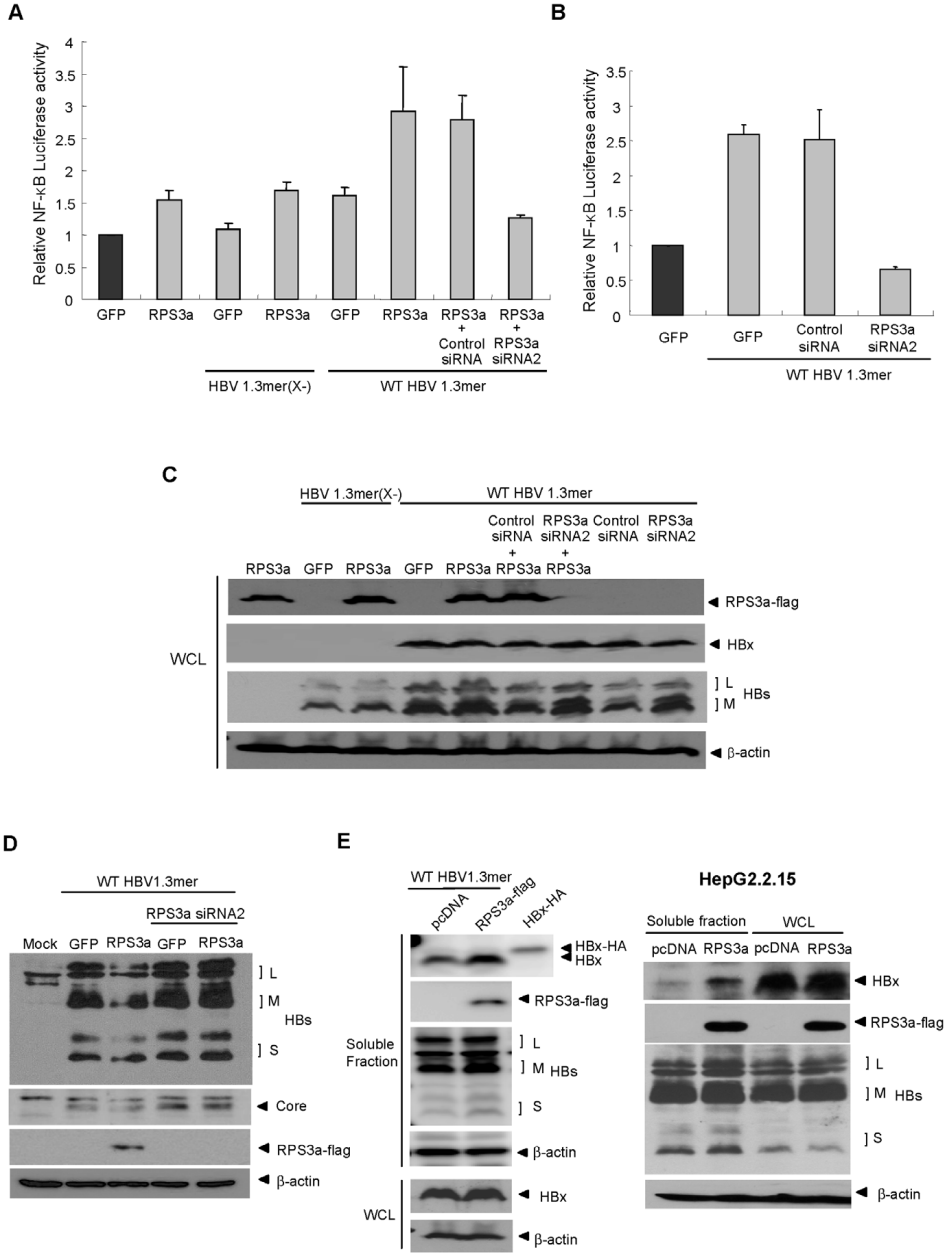
RPS3a enhances HBx-induced NF-κB signaling via its chaperoning function in the context of a replication-competent HBV genome. (**A**) RPS3a enhances the HBx-induced NF-κB activity in HBV full genome context. NF-κB activity was determined after co-transfection of the replication-competent wild-type HBV 1.3mer or HBx-deficient HBV 1.3mer(X-) with pcD-RPS3a-flag and pNF-κB-Luc in Huh7 cells. (**B**) Effect of endogenous RPS3a on HBV-mediated NF-κB activation. NF-κB activity was measured at 30 hr post co-transfection of wild type HBV1.3mer and pNF-κB-Luc with/without the indicated siRNAs in Huh7 cells. (**C–D**) Effect of over-expression and knock-down of RPS3a on genome-driven viral proteins. Cells were co-transfected as indicated in Figure 8A and 8B and harvested after 48 hr post-transfection. Western blot was performed using the whole cell lysate (WCL). Note that HBx protein was only detected in cells transfected with WT HBV1.3mer. The expression levels of HBV proteins (HBs and core protein) were constant under the over-expression or knock-down of RPS3a. L, M and S represent large, middle and small surface antigens, respectively. (**E**) RPS3a increases the soluble expression of genome-driven HBx. Huh7 Cells were co-transfected with WT HBV 1.3mer and pcD-RPS3a-flag and harvested at 48 hr post-transfection. Whole cell lysates (WCL) and soluble fractions (supernatant of WCL after centrifugation) were analyzed by western blot by using indicated antibodies (left). HBx-HA was used as positive control for the specificity of anti-HBx antibody. HepG2.2.15 cells were transfected with pcD-RPS3a-flag and harvested at 48 hr post-transfection. WCL and soluble fractions are analyzed by western blot (right).

The evidence so far of RPS3a-mediated upregulation of HBx-induced NF-κB signaling through its chaperoning activity, is based on HBx over-expression system driven by a non-viral promoter. Therefore, to investigate whether the effects of RPS3a on HBx also work under more physiological condition, we used a replication-competent full wt HBV genome [wt HBV 1.3mer] and HBx-deficient HBV genome [HBV 1.3mer(X-)]. We also confirmed that neither over-expression nor knock-down of RPS3a affect the total levels of viral proteins (HBx, surface and core) which are expressed by own viral promoters (Figure 8C and 8D). The NF-κB luciferase assay showed that RPS3a exerts no effect on HBV 1.3mer(X-); however, RPS3a hyperactivated the NF-κB activity when co-transfected with wt HBV 1.3mer (Figure 8A). This result demonstrates that the enhanced NF-κB signal is also HBx-dependent in full HBV genome system. As in Figure 5C, the RPS3a-mediated NF-κB hyperactivation by genome-driven HBx was also abolished by siRNA against RPS3a. When endogenous RPS3a was depleted by siRNA, the HBV-meditated NF-κB activation was also dramatically decreased under the basal level (Figure 8B).

To investigate whether the over-expressed RPS3a enhances the soluble expression of genome-driven HBx, we employed the transient transfection of HBV1.3mer and HBV-stable cell line, HepG2.2.15 systems. The results also clearly show that the RPS3a enhances the soluble expression of HBx that is expressed by HBV genome. This phenomenon was more prominent in HepG2.2.15 cells (Figure 8E). Taken together; these data suggest that over-expressed RPS3a in HCC can enhance the HBx-induced NF-κB signaling in the natural course of HBV infection through the increase of functional activity of HBx.

Finally, we also examined if HBV or HBx could affect the expression of RPS3a. Neither HBV nor HBx (Figure 7C) affects the expression level of RPS3a in itself (data not shown), suggesting that the over-expression of RPS3a in HBV-associated HCC tissues is HBV-independent.

## Discussion

In the present study, we addressed the role of RPS3a, a protein over-expressed in the HBV-associated HCC, on the HBx-induced NF-κB activation as a critical factor for oncogenesis. Molecular studies revealed that RPS3a has a novel chaperoning activity and provides its chaperoning function to the highly aggregation-prone HBx. Although many of the host proteins have been well known to be over-expressed in the virus-caused cancers, their role for the viral pathogenesis remains unsolved. Our finding on the interplay between RPS3a and HBx gives some clues to the role of over-expressed host factors, especially in the HBV-associated HCC.

Here, the over-expressed proteins in the HBV-caused HCC [13], [20] were investigated to determine whether they have some relationship with the HBV pathogenic effects, especially the HBx-caused HCC development. Interestingly, among the over-expressed proteins, many of them were ribosomal proteins. Although the expression of ribosomal proteins would be expected to be higher in the rapidly growing tumors for ribosomal assembly and protein synthesis, the stoichiometric ratio between ribosomal proteins was unbalanced in tumors [13]. This finding indicates that the over-expressed ribosomal proteins can be involved in other biological roles besides protein synthesis.

Most of the over-expressed ribosomal proteins activated the HBx-induced NF-κB signal; among them, RPS3a hyperactivated the HBx transactivity in the hepatoma cell line (Figure 1). And we showed that protein level of RPS3a is significantly higher in the HBV-related HCC tissues as compared with non-tumor tissues (Figure 2C). In addition, we found that NF-κB hyperactivation is closely linked to the soluble expression of otherwise aggregation-prone HBx (Figure 3, 4 and 8). Molecular studies have revealed that the insoluble granule-like punctate form of HBx was solubilized by interaction with the N-terminal domain of RPS3a (Figure 6 and 7). Therefore, we suggest that RPS3a provides chaperoning function to HBx preventing it from aggregation into punctate form. This was confirmed in both liver cells (Figure 4D and 4E) and the *E. coli* system (Figure 4F). In particular, RPS3a also enhanced the soluble expression of genome-driven HBx suggesting the physiological relevance of our finding (Figure 8E). Of the interest, the soluble expression of genome-driven HBx in HepG2.2.15 is very low, whereas the total expression is high. Until now, there is no clear evidence that HBx forms aggregates during HBV replication. Therefore, it is not clear whether the insoluble HBx is cytoplasmic insoluble aggregates or associated with nucleus or cytoplasmic organelles. However, our data clearly demonstrate that RPS3a solublizes the HBx into cytoplasm. Although the definite mechanism of RPS3a-mediated chaperoning effect and the biological relevance of our studies to a true HBV infection require further studies, it is likely that the solubility enhancement of HBx by RPS3a could be eventually responsible for the increased functional activity of HBx. The enhancement of HBx functional activity by RPS3a may subsequently amplify the known HBx-related oncogenic signaling pathways including NF-κB activation.

Interestingly, RPS3a has been associated with cell transformation, growth [18], [26], [27] and many cancers [20]–[25]. In particular, RPS3a is associated with EBV, an oncogenic virus [17]. Similar to HBx in our studies, EBNA-5, an EBV-encoded viral protein, binds with RPS3a and induces RPS3a expression, suggesting a role of RPS3a in EBV-induced tumorigenesis [17]. In this regard, the binding between HBx and RPS3a may contribute to the development and progression of HBV-induced HCC.

Many previous reports have shown that chaperones are associated with cancer development. Generally, chaperones such as HSP70 and HSP90, which are highly expressed under various stress conditions such as heat shock, hypoxia and cancer cells, stabilize abnormally folded or partially denatured proteins, and intrinsically unstable cellular and viral oncoproteins that play a key regulatory roles in signal transduction, cell survival, cancer development and apoptosis [38]–[40]. As an example, HSP90 is known as a molecular chaperone of numerous oncoproteins that accelerates cancer development by stabilizing the structure and function of those cellular factors, such as mutated p53, Bcr-Abl, Akt, Raf-1, Hif-1a and SRC tyrosine kinase [39], [40].

Recently, a potent extra-ribosomal chaperone activity of several ribosomal proteins, commensurate to well known chaperone (HSP90), was reported in *E. coli* [41]. In addition, over-expressed ribosomal proteins also functioned as chaperones in *Saccharomyces cerevisiae* [42]. Moreover, our recent reports have shown that soluble macro-molecules can intrinsically provide chaperoning function through interaction with aggregation-prone proteins [43]–[45]. These studies support our findings that RPS3a enhances the risk of cancer development by retaining the functional stability of viral regulatory HBx protein via chaperoning activity.

RPS3a has been reported to be highly expressed in most tumors including HCC [13], [20], thyroid carcinoma [21], virus-associated lymphoma [25], lung cancer [23], colorectal cancer [22] and thymic tumor [24]. However, the biochemical roles of RPS3a in these cancers have not been known yet. In this regard, RPS3a might be another class of molecular chaperone acting on cancer-related proteins including cellular and viral proteins. Here, we propose that abnormally over-expressed RPS3a in HBV-associated HCC might allow the structurally unstable viral HBx protein to gain the stability and functional activity by its chaperoning activity. HBx, having acquired stability and solubility by RPS3a, can then exert an effective viral oncogenic activity.

Our present results have many implications in HBV-associated liver diseases. HBx, a multifunctional viral protein, has been known to interact with a variety of cellular proteins involved in control of cell cycle, induction of proto-oncogenes, transactivation of key genes related to cell growth. Nevertheless, the extensive studies on the chaperoning roles of those interacting proteins on HBx have not been considered. Our results imply that HBx might gain its stability against aggregation by interaction with those cellular partners, which consequently lead to the HBx-related various pathogenic effects during HBV infection. In addition, HSP90 is already a potential anti-cancer drug target and numerous small molecule inhibitors of HSP90, such as geldanamycin, have been developed and used in conjunction with other anti-cancer drugs, for combinational therapies [40], [47]. Similarly, our study suggests that inhibition of cellular partners from endowing the functional stability for HBx could be another target for the prevention of HBV-associated liver diseases.

## Materials and Methods

### Plasmids construction

The details on HBx (subtype *ayw*) and HBx-GFP clones have been described previously [32]. The expression plasmid for wild-type HBx-HA was generated by PCR in pcDNA3.1 vector (Invitrogen). The wild-type HBV 1.3mer and HBx-deficient HBV 1.3mer(X-) plasmids were kindly provided by Professor WS Ryu (Yonsei University). The cDNAs of Hsp70p2, RPL27a, RPS20, RPL8, RPS24, RPS27a, RPL21, RPL35a and RPS3a were obtained from Korean UniGene Information (KUGI) and sub-cloned into pcDNA3.1. Additionally, the wild-type, deletion mutants (M1, M2, M3, M4, M5) of RPS3a-flag and RPS20-flag were generated by PCR. For expression in *E. coli*, pGE-RPS3a-His, pBad-RPS3a-His, pBad-RPS20-His, pBad-EGFP-His and pT7-HBx-HA were constructed by PCR using NdeI and HindIII restriction enzyme sites.

### Reagents and Cell transfection

Antibody for the detection of GFP was purchased from Clontech (Palo Alto, CA). Antibodies for α-tubulin was purchased from Santa Cruz Biotechnology (Santa Cruz, CA) and those for lamin A/C and NF-κB p65 were obtained from Cell Signaling (Danvers, MA). To detect the HBV surface, core and HBx proteins, the anti-HBV surface antigen (abcam, MA, USA), anti-HBV core (Dako, Denmark) and the polyclonal anti-HBx (MYBiosource, CA, USA) antibodies were used, respectively. The anti-Flag M2 monoclonal antibody, rabbit anti-HA, anti-β-actin and secondary antibodies were obtained from Sigma (St. Louis, MO). Alexa Fluor-568 secondary antibody was purchased from Invitrogen (Carlsbad, CA). The human hepatoma cell lines (Huh 7, HepG2 and HepG2.2.15 cell lines) were obtained from the American Type Culture Collection (ATCC, Manassas, VA). Huh7, HepG2 and HepG2.2.15 were maintained in DMEM (Gibco BRL, Oregon, USA) supplemented with 10% heat-inactivated FBS and 1% antibiotic-antimicotics (Gibco BRL, Oregon, USA). Transient transfections (80%∼90% cell confluency) were performed using Lipofectamine2000 (Invitrogen, Carlsbad, CA) or LT1 (Mirus, Madison, WI) according to the manufacturer's instruction.

### Luciferase assay

Luciferase assays were performed for the detection of HBx or HBV genome-mediated NF-κB activation. Approximately 4×10^4^ Huh7 and HepG2 cells were seeded into 12-well culture plates, and transiently transfected with a DNA mixture containing 1.05 µg of plasmid (0.25 µg pNF-κB-Luc, 0.4 µg HBx or HBV 1.3mer, and 0.4 µg of test plasmids). To normalize the total amount of transfection DNA, empty vector (pCMV) was used as needed. Cells were harvested and lysed at 36 hr after transfection (otherwise mentioned in Figure legends) and assayed for luciferase activity using the Steady Glo-Luciferase system (Promega, Madison, WI). Data were collected from the results of at least five independent experiments. The statistical significance was verified by statistical t-test analysis (P<0.05).

### Western blot and immunoprecipitation assay

Western blot analyses and immunoprecipitation assays were carried out as described previously [32]. Cells were lysed using lysis buffer [25 mM Tris/HCl, 1% NP-40 (or 1% Triton X-100 for whole cell lysate), 0.5% protease inhibitor cocktail (Sigma, St.Louis, MO), 150 mM NaCl, 2 mM KCl, pH 7.4]. For immunoprecipitation, cells were lysed with 0.3 ml or 0.4 ml of immunoprecipitation lysis buffer [25 mM Tris/HCl, 1% Triton X-100 (or 0.5% NP-40), 0.5% protease inhibitor cocktail, 150 mM Nacl, 2 mM KCl, pH 7.4]. The clarified total cell lysates were incubated with primary antibodies for 24 hr at 4°C with gentle inverting and then mixed with 10 µl of protein-A agarose (Roche, Mannheim, Germany) followed by overnight incubation at 4°C. After washing in PBS three times, the immune complex was mixed with 5× SDS sample buffer. After blotting, membranes were stripped with stripping buffer (Pierce, Rockford, IL) and reblotted.

### Fluorescence analysis

To observe punctate expression of HBx, fluorescence analysis was performed as previously described [32]. Huh7 cells were co-transfected with pEGFP or pEG-HBx-GFP plasmid and test plasmids. After 36 hr, the cells were analyzed by fluorescence microscopy (magnification, ×200). For the co-localization assay, RPS3a-flag and HBx-GFP plasmids were co-transfected in Huh7 cells grown on cover glass to 50% confluency. At 24 hr post-transfection, the cells were fixed with fixing solution (methanol/formaldehyde = 99∶1). The cells were permeabilized with 0.2% Triton X-100 for 30 min on ice. PBS containing 1% BSA was used for blocking at room temperature for 1 hr and cells were washed with PBS. The cells were incubated with anti-flag antibody (1∶50) overnight at 4°C. After washing, cells were incubated with 1∶1000 diluted Alexa Fluor-568 goat secondary antibody for 40 min at room temperature. After 3 washes with PBS, DAPI solution (1 µg/ml) was used to stain the nucleus. Cells were mounted and examined under a fluorescence microscope (magnification, ×200).

### Generation of RPS3a antibody using recombinant protein

Rabbit polyclonal antibodies for detection of RPS3a were generated using full-length recombinant human RPS3a proteins expressed in *E. coli*. First, human RPS3a with 6× His tag was cloned in *E. coli* expression pGE vector, controlled by T7 promoter and protein was over-expressed in BL21(DE3)pLysS *E. coli* strain (Novagen). Recombinant RPS3a-His proteins were solubly expressed in *E. coli*. For purification of recombinant RPS3a-His protein from *E. coli* extracts, one-step Ni-affinity chromatography was performed twice using AKTA Prime FPLC (GE Healthcare). Rabbits were immunized with the purified recombinant RPS3a-His proteins, and the 1^st^ and 2^nd^ boosts were administered at 4 weeks and 6 weeks after the primary immunization. The polyclonal anti-RPS3a antibodies were purified from serum using protein A beads. Antibody affinity for RPS3a protein was tested by ELISA and western blot analysis. The generated rabbit polyclonal anti-RPS3a antibodies could detect the endogenous human and mouse RPS3a.

### Immunohistochemistry

The 20 pairs of HBV-positive human liver tissues consisting of non-tumor and tumor regions were obtained from ISU Abxis (Seoul, Korea). The information on liver tissues is summarized in Table 1. Liver sections were rehydrated using graded alcohols. For antigen retrieval, pretreatment in citrate buffer (pH 6.0) was performed. Endogenous peroxidase was inactivated with methanol containing 3% H_2_O_2_. After blocking, the tissue slide was incubated overnight with rabbit polyclonal anti-RPS3a antibody (generated in our lab) in a wet chamber at 4°C. After washing with PBS, the tissue slide was incubated with goat anti-rabbit antibody (EnVision™ Kits, DAKO, Denmark) at room temperature. The slides were developed using 3,3′-diaminobenzidine tetrahydrochloride chromogen solution (DAKO). The slides were then counter-stained with hematoxylin.

### RT-PCR and RNA interference experiment

Total RNAs were isolated using Trizol solution (Invitrogen, Carlsbad, CA) according to the manufacturer's protocol. Using 2 µg of total RNA and oligo dT primer, first-strand cDNA was synthesized by the Power cDNA synthesis kit (Intron Biotechnology, Seoul, Korea). A mixture of 1 µl cDNA product, 0.5 mM target specific primers (RPS3a, GAPDH), 0.5 U ex*Taq* polymerase (Takara, Shiga, Japan) and 0.25 mM dNTP were used for PCR (24 cycles). The specific primers were as follows: RPS3a sense, 5′-GTC ACG CTC GAG ATG AGG ACC CAA GGA ACC-3′ and antisense, 5′-GTC ACG AAG CTT TTA AAC AGA TTC TTG GAC-3′; GAPDH sense, 5′-CGT CTT CAC CAC CAT GGA GA-3′ and antisense, 5′-CGG CCA TCA CGC CAC AGT TT-3′. After resolving the products on 1.5% agarose gels, the relative expression levels were estimated using Bio-1D image analysis software (Vilber Lourmat).

For knock-down studies of RPS3a, siRNAs against RPS3a were synthesized by Samchully Pharm (Seoul, Korea). Sense and antisense RNA fragments were mixed with 5× universal binding buffer and incubated at 90°C for 2 min. For annealing, the mixture was reincubated for 1 hr at 30°C. The details of siRNA sequences are follows: RPS3a siRNA1 sense, 5′-GUG CUA AAG UUG AAC GAG C -3′ and antisense, 5′-GCU CGU UCA ACU UUA GCA C -3′; RPS3a siRNA2 sense, 5′-GGA UCU UAC CCG UGA CAA A -3′ and antisense, 5′-UUU GUC ACG GGU AAG AUC C -3′; RPS3a siRNA3 sense, 5′-AGA UUG GUA UGA UGU GAA A -3′ and antisense, 5′-UUU CAC AUC AUA CCA AUC U -3′; control siRNA lamin sense, 5′-CUG GAC UUC CAG AAG AAC ATT-3′ and antisense, 5′-UGU UCU UCU GGA AGU CCA GTT-3′; GL3 siRNA sense, 5′-CUU ACG CUG AGU ACU UCG ATT-3′ and antisense, 5′-UCG AAG UAC UCA GCG UAA GTT-3′; GFP siRNA sense, 5′-GUU CAG CGU GUC CGG CGA GTT-3′ and antisense, 5′-CUC GCC GGA CAC GCU GAA CTT-3′. Transfection of siRNA was performed using Lipofectamine 2000 (Invitrogen, Carlsbad, CA). The effects of RPS3a siRNAs were verified by western blotting.

### Electrophoretic mobility shift assay (EMSA) and NF-κB EMSA-ELISA analysis

At 18 hr post-transfection, cells were harvested and the nuclear and cytoplasmic fractions were prepared using Nuclear and Cytoplasmic Extraction Reagents (Pierce, Rockford, IL). The level of p65 translocation into nucleus was measured by EMSA-ELISA analysis (EZ-Detect Transcription Factor Kits for NF-κB p65, Pierce, Rockford, IL) and EMSA assay. For EMSA-ELISA analysis, the nuclear extracts (4 µg) were incubated with the supplied plate and the level of p65 was determined by ELISA. For EMSA, the nuclear extracts (3 µg) were pre-incubated with 2 µg of poly(dI-dC) for 10 min in total 20 µl reaction buffer [10 mM Tris-Cl(pH 7.5), 100 mM Nacl, 1 mM EDTA, 0.5 mM DTT, 10% glycerol]. After pre-incubation, the [^32^P]-end-labeled dsDNA oligonucleotide (NF-κB consensus oligonucleotide: 5′-AGTTGAGGGGACTTTCCCAGGC-3′, Promega, Madison, WI) was added and incubated for 15 min. After the binding reaction, DNA-protein complex was analyzed by electrophoresis at room temperature using 5% polyacrylamide gel containing 10% glycerol and 1× TBE. Then, gels were dried and autoradiographed. For the competition assay, unlabeled NF-κB oligonucleotide was added and incubated for 25 min before treatment with [^32^P]-labeled probe. Each analysis was carried out at least 5 times.

### Solubility enhancing experiment in *E. coli* system

For the induction of proteins in *E. coli*, the pT7-HBx-HA (Kan+) and pBad-RPS3a-His(Amp+) clones, which were inducible by IPTG and L-Arabinose, respectively, were constructed. Both plasmids were co-transformed in BL21(DE3)pLysS *E. coli* strains with LB media containing chloramphenicol, kanamycin and ampicillin. The RPS3a-His and HBx-HA proteins were induced at 30°C by treatment with 0.2% L-Arabinose and 1 mM IPTG (Sigma, St.Louis, MO), respectively. After induction, cells were cultured for 3 hr at 20°C and harvested by centrifugation at 3000 rpm. Cells were lysed by sonication and divided into three fractions [total lysate (T), supernatant (S), and pellet (P)] by centrifugation. As the controls, pBad-RPS20-His(Amp+) or pBad-EGFP-His(Amp+) were used for co-transformation with pT7-HBx-HA (Kan+). Detailed solubility analysis was performed as described previously [45], [46]. The solubility of HBx-HA, *i.e.*, the relative ratio of (S)/(T), was estimated by Bio-1D image analysis software (Vilber Lourmat).
